# The Potential of QP3VH-Chitosan Peptide as Biomimetic Remineralization in Early Dental Caries Treatment: An *In Vitro* Study

**DOI:** 10.1055/s-0044-1782189

**Published:** 2024-05-17

**Authors:** Prima Agusmawanti, Diatri Nari Ratih, Nunuk Purwanti, Tri Joko Raharjo

**Affiliations:** 1 Doctoral Program, Faculty of Dentistry, Universitas Gadjah Mada, Yogyakarta, Indonesia; 2 Departement of Pediatric Dentistry, Faculty of Dentistry, Universitas Islam Sultan Agung, Indonesia; 3 Department of Conservative Dentistry, Faculty of Dentistry, Universitas Gadjah Mada, Yogyakarta, Indonesia; 4 Department of Dental Biomedical Sciences, Faculty of Dentistry, Universitas Gadjah Mada, Yogyakarta, Indonesia; 5 Department of Chemistry, Faculty of Mathematics and Natural Sciences, Universitas Gadjah Mada, Yogyakarta, Indonesia

**Keywords:** biomimetic, remineralization, caries, QP3VH peptide, chitosan, NaF

## Abstract

**Objectives** The development of remineralization biomimetics using organic peptide molecules is expected to resemble the hydroxyapatite (HA) mineralization process in tooth enamel. The development of an amelogenin derivative peptide combined with antimicrobial peptide was designed, resulting in QP3VH. This combination then was mixed with chitosan as a carrier. This study aimed to evaluate the biomimetic efficacy of QP3VH as a remineralizing agent combined with chitosan.

**Materials and Methods** Fifty deciduous mandibular incisor enamel samples were used in this study. The artificial enamel lesions were created on a buccal surface and were randomly assigned to five groups of 10 each according to the remineralizing agent used: QP3VH, NaF, QP3VH + NaF, QP3VH + CS (QP3VH + chitosan), and saline distilled water (SDW). Each group was performed pH cycling for seven days. Enamel surface morphology and evaluation of mineral content Ca/P (calcium and phosphate) using scanning electron microscopy and energy dispersive X-ray analysis. The assessment was carried out, after demineralization, and after application with remineralization agents.

**Statistical Analysis** Data were analyzed using a one-way analysis of variance followed by least significance difference post-hoc test. The paired *t* -test was utilized to compare the demineralization and remineralization results. The significance level used was 95%.

**Results** The remineralized group exhibited a significant increase in calcium and phosphate content on the enamel surface ( *p* <0.05), and QP3VH + CS produced the maximum Ca/P mass percent after remineralization.

**Conclusion** Combining QP3VH with chitosan produces greatest remineralization than QP3VH, QP3VH + NaF, Naf, and SDW; therefore, QP3VH peptide has potential as a remineralizing agent, in the future

## Introduction

Caries is a common dental and oral health issue affecting infants and adults. Dental caries affects 60 to 90% of school-aged children worldwide. 1 2 One or more of the complicated diseases is known as early childhood caries, which is the presence of one or more caries-related missing, filled, or decayed (noncavitated or cavitated lesions) tooth surfaces in any primary tooth in a child under the age of 6 and usually begins with white spot lesions. The early stages of tooth decay or demineralization, known as incipient carious lesions, which are smooth surface caries and active lesions limited to the enamel with formation of a chalky white spot on the tooth's surface, which represents an area of enamel demineralization, is the initial indication of a new carious lesion. 3

In pediatric dentistry, a minimally invasive approach with remineralization therapy has been popular for treating incipient lesions. 4 Remineralization can be stimulated by various agents, such as inorganic and organic compounds (peptides). 5 6 7 Recently, a minimally invasive topically fluoride method has been effective. However, the disadvantage of fluoride is that it is only effective on surface lesions no deeper than 30 µm. 8 The efficacy of fluoride is dose dependent; hence, increasing the dose is limited since it can cause fluorosis. The recommended fluoride concentration in children's toothpaste is between 1,000 and 1,500 ppm; it is optimal for effectively remineralization early caries lesions. 9 Researchers have been attempting to find an alternative material that could be beneficial for remineralization agents without the possible risk associated with fluoride. 7

Previous investigators have studied to develop bioactive additives that increase enamel remineralization in a biomimetic approach. 9 Several biomimetic peptides containing amelogenin residues have the potential for enamel remineralization. 10 Mineralization is known as to synthesize a new peptide comprised only of functional fragments of the N-terminus and C-terminus of amelogenin, which promote remineralization in enamel caries lesions; this method is based on the theory of the apatite binding and mineralization in amelogenin. 11

This study has been conducted to design and synthesize bifunctional peptides to control early tooth caries by developing peptides that have dual-action system functions. The system can simultaneously generate cariogenic processes, such as the anticaries peptide consisting of remineralization and antimicrobial agents. 10 In this study, combining a peptide derivative of amelogenin QPX3 (QPHQPMQPQ) has more significant potential in increasing the degree of hydroxyapatite orientation, where the middle area of amelogenin consisting of polyproline is associated with the formation of hydroxyapatite crystals. 12 It was then combined with an antimicrobial peptide, namely TVH19 (TKRQQVVGLLWHLLHHLLH-NH2) that has high antibacterial abilities against cariogenic bacteria and remineralization capabilities, 13 resulting in a new peptide, named QP3VH (QPHQPMQPQTKRQQVVGLLWHLLHHLLH- NH2).

Moreover, chitosan can prevent hydrogen ions from reaching the mineral surface by forming a protective layer on the enamel and ensuring electrostatic interaction with the peptide, thereby preventing its solubility. 14 This study aimed to evaluate the QP3VH peptide in conjunction with several other mineral agents whose function would be enhanced by adding NaF and chitosan as mineral transport carrier materials that play a role in remineralizing early caries lesions.

## Materials and Methods

The research protocol has been approved by institutional ethics committee Faculty of Dentistry, Universitas Gadjah Mada Yogyakarta, Indonesia, under the number 0030/KKEP/FKG-UGM/EC/ 2022. In this study, three remineralizing agent constituents were evaluated: the QP3VH peptide based on amelogenin (GL Biochem, Shanghai, China) and consists of the residues (QPHQPMQPQTKRQQVVGLLWHLLHHLLH- NH2). Then it was purified via reverse-phase high-performance liquid chromatography and was characterized by mass spectrometry. Chitosan with 75 to 85% deacetylation was used in this study (Sigma-Aldrich, Michigan, United States). QP3VH + chitosan was made as described with some modifications. In total, 1 mL of a chitosan calcium phosphate solution (960 mL of 1% chitosan, 25 mL of 0.1 M CaCl _2_ , and 15 mL of 0.1 M Na _2_ HPO _4_ ) was mixed with QP3VH to obtain the final concentration of 50 µM, followed by stirring 8 hours at a temperature of 23°C. The pH value was adjusted to 6.0 by using diluted NaOH 15 and NaF 1,000 parts per million (ppm).

The extracted human mandibular first primary incisors, which are intact, free of caries, and devoid of anomalies, were used in this study. A cylinder-shaped plastic mold was prepared to create the sample block, then self-curing acrylic resin was placed into the mold, and the tooth was implanted with its buccal surface faced up. All teeth were assigned randomly into five groups of ten each—Group 1: Application with QP3VH peptide; group 2: with 1,000 ppm NaF; group 3: with QP3VH peptide+ 1,000 ppm NaF +; group 4: with QP3VH peptide+ chitosan, and group 5: with sterile distilled water (SDW). The buccal surface enamel of each sample from each group was applied with each remineralization agent with a volume of 20 µL using a micropipette for 5 minutes twice a day, before and after the demineralization.

### Creation of Initial Caries Lesion

In the demineralization treatment, enamel initial caries lesions were created by applying a gel of 37% phosphoric acid to the surface of the enamel for 60 seconds. 16

### pH Cycling

The pH cycling was done to stimulate the buccal cavity in oral environment. Each samples wash carried out with a pH cycle for 7 days. Each sample block was stored in demineralization solution (2 mM CaCl _2_ ·2H _2_ O, 2 mM KH _2_ PO _4_ , 50 mM sodium acetate, and 0.879 mL acetic acid adjusted at pH 4.6) for 3 hours (each volume for each sample block = 20 mL), and in remineralization solution (1.2 mM CaCl _2_ ·2H _2_ O, 0.72 mM K _2_ HPO _4_ , 16 mM KCl, 0.2 mM MgCl _2_ ·6H _2_ O, 50 mM HEPES, and 4.5 mM NH _4_ Cl in SDW adjusted to pH 7.2) for 20 hours (each volume for each sample block = 10 mL). After each treatment, the samples were rinsed with deionized water. This cycle was repeated every day for 5 days, and after 5 days, the sample remained in the remineralization solution for 2 days before observation using scanning electron microscope (SEM). All samples were stored in an incubator with temperature of 37°C. 17

### Scanning Electron Microscope—Energy Dispersive X-Ray Analysis

After demineralization and remineralization, the enamel morphology was observed using SEM (JEOL JSM IT- 200, Tokyo, Japan) at 1000x, and 5000x magnifications. All samples were subjected to evaluation of mineral content (mass/atomic percentage) using EDX. The degree of remineralization was determined by quantifying the amount of Calcium (Ca) and phosphate (P) in each sample. The digital outputs of the EDX values were interpreted numerically as Ca/P ratios.

### Statistical Analysis

Statistical analysis was performed with IMB SPSS (version 26) software (SPSS Inc. IBM Corporation, New York, United States). Previously, the normality and homogeneity of the data were evaluated. The Shapiro–Wilk test was used to determine the normality of the data obtained. The homogeneity test employed Levene's variance. The Shapiro–Wilk test produced a normal distribution, and Levene's variance generated data were homogeneous. A one-way analysis of variance (ANOVA) was performed followed by a least significance difference post-hoc test. The paired *t* -test was utilized to compare the demineralization and remineralization results. The level of significance used was 95%.

## Results

**Fig. 1:**
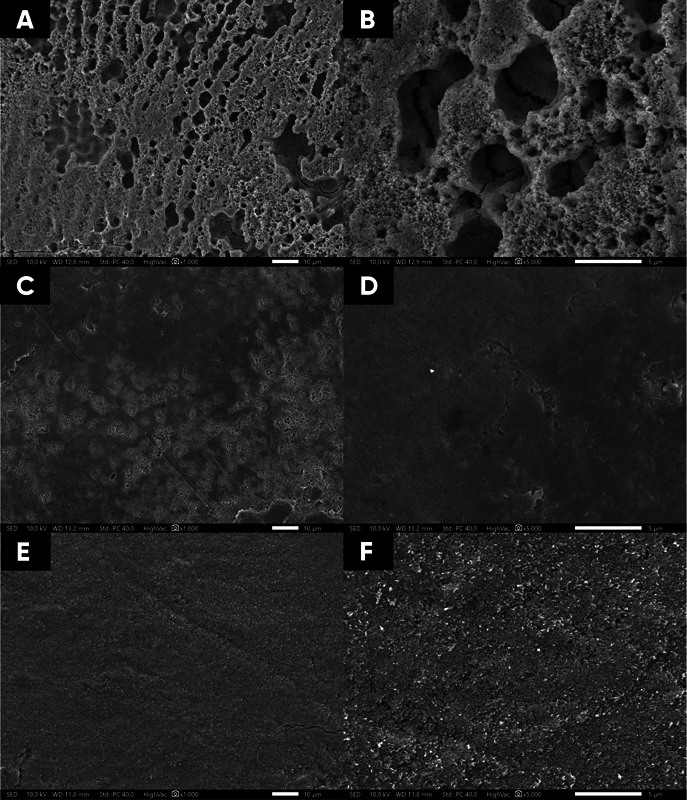
Scanning electron microscopy photomicrographs representative of the tooth enamel surface. Demineralized enamel was treated with 37% phosphoric acid ( **A** , **B** ) and after treated with the remineralizing agent QP3VH peptide ( **C** , **D** ) and with NaF 1,000 ppm ( **E** , **F** ). 1000× magnification ( **A** , **C** and **E** ) and 5000× magnification ( **B** , **D** , and **F** ).

SEM showed that the remineralization and demineralization groups produced different morphological images. After demineralization with 37% acid etching, an unequal, irregular, and rough surface with increased porosity resembling a honeycomb structure is shown in Fig. 1A and B . This surface was characterized by the dissolution of apatite crystals in the prism, which resulted in the loss of aprismatic enamel. In contrast, remineralization with QP3VH revealed that the surface of the interprismatic and prismatic enamel was covered by a densely, smooth, uniform homogeneous layer as well as decrease in porosity ( Fig. 1C, D ). The group applied with NaF ( Fig. 1E, F ) showed that the surface was scattered with microparticle deposits and the apertures were coated with a slightly rough surface.

**Fig. 2:**
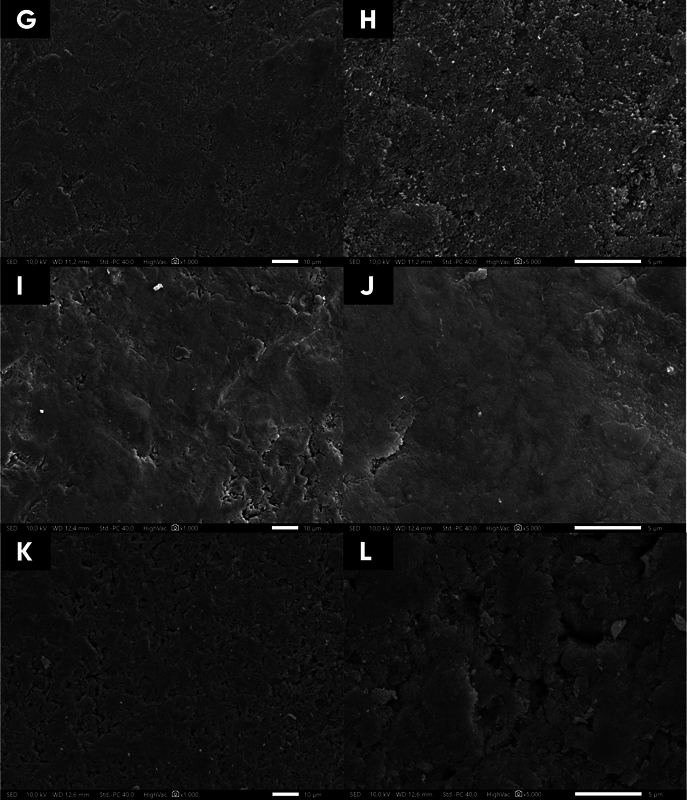
Scanning electron microscopy photomicrographs representative of the tooth enamel surface after treatment with the remineralizing agent QP3VH peptide + NaF 1000 ppm ( **G** , **H** ), with QP3VH peptide + chitosan ( **I** , **J** ) and with saline distilled water ( **K** , **L** ). 1000× magnification ( **G** , **I** and **K** ) and 5000× magnification ( **H** , **J** and **L** ).

After combining NaF and QP3VH ( Fig. 2G, H ), the images exhibited the presence of dense deposits, indicating a surface with particle deposits covering slightly smaller pores occurred. Applying QP3VH + chitosan ( Fig. 2I, J ) produces the smoother enamel surface; the interprismatic enamel structure was visible and covered by a thick and uniform homogeneous layer. On the other hand, the control group (SDW) ( Fig. 2K, L ) demonstrated that depression was visible, indicating that the depth of porosity was still wide even though it was slightly smaller.

**Fig. 3:**
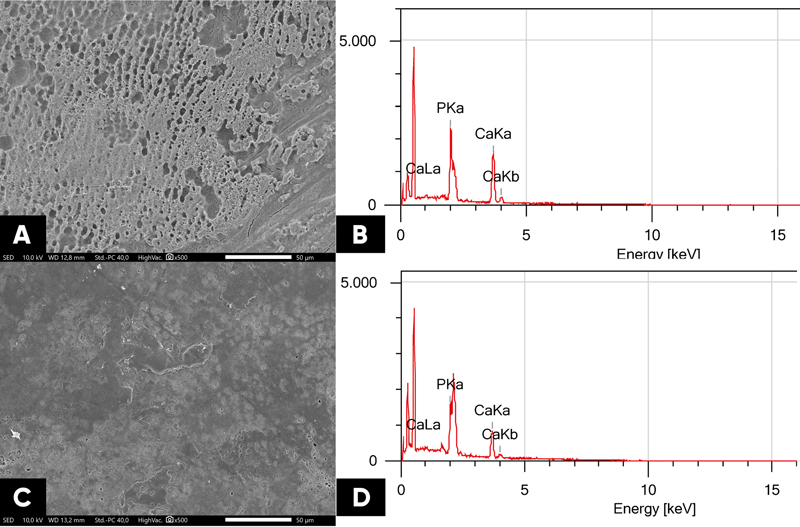
Scanning electron microscopy photomicrographs representative of the tooth enamel surface of demineralized ( **A** ) and post-remineralized QP3VH peptide ( **C** ). Energy dispersive X-ray analysis interpretation of demineralized ( **B** ) and post-remineralized QP3VH peptide ( **D** ).

Remineralization using QP3VH peptide revealed the alterations in the enamel surface morphology. The SEM image of samples after demineralization using 37% etching generated an enormous microporosity with a rough enamel surface resembling a beehive and the loss of aprismatic enamel ( Fig. 3A ). On the contrary, SEM images of the remineralized enamel surface reveal an improvement in surface quality, with increasingly homogeneous amorphous deposits and closed fissures ( Fig. 3C ). Compared to demineralization ( Fig. 3B ), the mass percentage of Ca/P increased following remineralization ( Fig. 3D ).

Data of Ca/P ratio after remineralization were analyzed using the one-way ANOVA which revealed that a significant difference occurred in the increased of the Ca/P ratio after application of the remineralization agent ( *p* < 0.05) that are presented in Table 1 . The post-hoc test analysis for each treatment group demonstrated the QP3VH + chitosan group produced the largest difference relating the mean value of Ca/P ratio between demineralization and remineralization ( *p* < 0.05) as shown in Table 2 . The paired *t* -test for comparisons after demineralization and remineralization showed the ratio of Ca/P values following remineralization was statistically significantly higher for all treatment groups and QP3VH+chitosan had the highest remineralization compared to the other groups. ( Table 3 ).

**Table 1:** One-way ANOVA of Ca/P ratio on the enamel surface after demineralization and remineralization

	Sum of squares	df	Mean square	F	Sig. ( *p-* value)
Between groups	7.423	9	0.825	87.201	0.000 a
Within groups	0.378	40	0.009		
Total	7.802	49			

Abbreviations: ANOVA, analysis of variance; Ca/P, calcium/phosphate.

a *p* -Value is significant at 0.05 level.

**Table 2:** LSD post-hoc test comparison for agent remineralization QP3VH, NaF, QP3VH + NaF, QP3VH+ chitosan and SDW based on Ca/P ratio value

(I) Group	(J) Group	Mean difference (I–J)	Std. error	Sig. ( *p* -value)	95% Confidence interval	
					Lower bound	Upper bound
QP3VH	NaF	−0.29800 a	0.08221	0.002 a	−0.4695	−0.1265
	QP3VH+ NaF	−0.17600 a	0.08221	0.045 a	−0.3475	−0.0045
	QP3VH+ chitosan	−0.69200 a	0.08221	0.000 a	−0.8635	−0.5205
	SDW	0.06200	0.08221	0.460	−0.1095	0.2335
NaF	QP3VH+ NaF	0.12200	0.08221	0.153	−0.0495	0.2935
	QP3VH+ chitosan	−0.39400 a	0.08221	0.000 a	−0.5655	−0.2225
	SDW	0.36000 a	0.08221	0.000 a	0.1885	0.5315
QP3VH+ NaF	QP3VH+ chitosan	−0.51600 a	0.08221	0.000 a	−0.6875	−0.3445
	SDW	0.23800 a	0.08221	0.009 a	0.0665	0.4095
QP3VH+ chitosan	SDW	0.75400 a	0.08221	0.000 a	0.5825	0.9255

Abbreviations: Ca/P, calcium/phosphate; LSD, least significance difference; SDW, saline distilled water.

a *p* -Value is significant at 0.05 level.

**Table 3:** Intragroup comparison for QP3VH, NaF, QP3VH + NaF, QP3VH+ chitosan and SDW, after demineralization(demin) and remineralization (Remin) based on Ca/P ratio value

Groups	Ca/P mass %	Mean	Std deviation	Paired differences		*p* -Value
				Mean differences	Std deviation	
**QP3VH**	Demin mass %	1.6880	0.06648	−0.75600	0.22722	0.002 a
	Remin mass %	2.3960	0.23650			
**NaF**	Demin mass %	1.6240	0.05030	−0.78800	0.11498	0.000 a
	Remin mass %	2.4120	0.12834			
**QP3VH + NaF**	Demin mass %	1.6400	0.05099	−0.66600	0.04722	0.000 a
	Remin mass %	2.3060	0.04506			
**QP3VH + chitosan**	Demin mass %	1.5200	0.05244	−1.18200	0.08556	0.000 a
	Remin mass %	2.7020	0.08258			
**SDW**	Demin mass %	1.7280	0.04970	−0.43600	0.07635	0.000 a
	Remin mass %	2.1640	0.08503			

Abbreviations: Ca/P, calcium/phosphate; SDW, saline distilled water.

a *p* -Value is significant at 0.05 level.

## Discussion

Based on a minimally invasive approach, early carious lesions are treated noninvasively through remineralization to prevent or reverse the progression of caries. The objectives of noninvasive treatment are caries prevention and shifting the equilibrium toward remineralization by reducing the solubility of hard tissue or increasing the surface area for mineral redeposition. Biomimetic remineralization strategies have gained increasing interest as anticaries treatments in recent years. 18 Previous studies have developed new bioactive agents, including peptide-based agents, for caries management. 19

A recent study has investigated the use of the QP3VH peptide as a remineralizing agent in conjunction with other remineralizing agent. The combination of SEM-EDX was utilized in this study to measure the integrity of the crystal structure and the Ca/P ratio. The SEM was also used to observe the morphology of the enamel surface qualitatively and to describe the ultra-morphological changes induced by various remineralizing agents. However, EDX was utilized to measure the integrity of the crystal structure and the Ca/P ratio. 20 The 7-day pH cycling was employed to investigate the effects of caries prevention agents on the demineralization and remineralization dynamics of enamel to simulate oral cavity environment. 17

In recent years, researchers have focused on protein/peptide modulated enamel biomimetic mineralization, 5 21 which can be exploited for the development and design of biomimetic materials with applications in biomedicine and dentistry. 22 Various peptides have been synthesized to repair damaged enamel by the role of enamel's matrix proteins in tooth formation. 23 QP3VH is a combination of the amelogenin-derived QPX3 peptide (QPHQPMQPQ) and the TVH19 peptide (TKRQQVVGLLWHLLHHLLH -NH 2), which possess potent antibacterial and remineralizing properties. 13 This study used peptides derived from natural enamel matrix proteins involved in the biomineralization process to develop a promising biomaterial for the remineralization of enamel.

It has been proven by SEM images that treating early caries lesions with the amelogenin-derived peptide remineralization agent QP3VH can increase mineral content by forming a three-dimensional scaffold that allows crystal deposition and growth. In accordance with the study by Xiang et al, 24 this study determined that synthesizing amelogenin and its derivative peptides promote the remineralization of early enamel caries lesions. Based on the highly conserved Gln-Pro-X repeats in amelogenin, this scaffold secures hydroxyapatite and promotes mineralization. 14 In addition, the synthesis of this peptide is enriched with proline, whose carboxyl group enhances its interaction with calcium and phosphate ions, thereby promoting hydroxyapatite crystallization. 25

According to Wang et al, 13 Transversal Microradiography (TMR) data analysis revealed substantially greater mineral deposition and shallower lesions in the groups treated with TVH19 or NaF. TVH19 reduces mineral loss to the same extent as NaF, and both agents generate lesions of comparable depth. This phenomenon demonstrates that TVH19 is capable of remineralization.

Fluoride continues to be used for caries prevention today. Fluoride is capable of forming fluorapatite crystals that are more acid-resistant and promote remineralization by attracting calcium and phosphate ions, thereby increasing the mineral content. In high concentration, fluoride increases the remineralization of the outer enamel and decreases the demineralization of the inner enamel, resulting in a superficial remineralization layer of the lesion body. 26 Extremely high fluoride concentrations are harmful, and fluorosis can result from levels even marginally above recommended values. 7

Organic compounds such as amelogenin can reduce the occurrence of disordered structures in a reaction system. Due to the limitation of fluoride, a strategy is required to combine fluorine with other compounds, such as peptides. Several studies have developed biomimetic enamel remineralization-increasing bioactive additives to find alternative materials to fluoride. 9

The SEM images of the NaF group combined with QP3VH showed that the surface of the structure was interspersed with microparticle deposits that covered the pores evenly with particle deposits and covered the pores that were smoother and thicker compared to the application with NaF alone. Similarly, QP3VH combined with chitosan, the interprismatic, and prismatic enamel surfaces appears covered by a thick, evenly smooth and uniform homogeneous layer, and the porosity decreases. EDX analysis results showed that the percentage of Ca and P recovered after remineralization, and QP3VH and chitosan produced the most significant percentage of Ca and P compared with other agents.

Ren et al 27 demonstrated the efficacy of chitosan and peptides in remineralization, where peptides regulate the growth of hydroxyapatite crystals and chitosan functions as a carrier with antibacterial activity to stabilize peptides. On the other hand, peptides in chitosan hydrogel capture calcium and phosphate from the environment and direct them to sequentially deposit on the demineralized enamel surface, thus accelerating remineralization. The high nitrogen content of chitosan makes it a prospective carrier substance for transporting biomineralization-related ions such as calcium and phosphate. 14 In addition, by forming a protective layer on the enamel and guaranteeing electrostatic interactions with beta sheet-structured peptides, chitosan can prevent hydrogen ions from penetrating the mineral surface. Interaction between peptide and chitosan can prevent the diffusion of hydrogen ions to the surface of the mineral by forming a protective layer on the enamel. 28

Despite the encouraging outcomes of this study, it is essential to remember that SEM-EDX only provides data regarding the morphology and elemental composition of the surface. It would also be interesting to learn more about the remineralization depth compared to remineralization, whether or not there are subsurface porosities and the properties of the remineralized layer.

## Conclusions

Combining QP3VH and chitosan produces the highest remineralization compared to the QP3VH, QP3VH + NaF, Naf, and SDW groups. The QP3VH peptide can be employed to treat incipient lesions in the future since this agent has the potential for remineralization, although several aspects need to be evaluated.
